# PD-L1 Expression and Clinicopathologic Associations in Mediastinal Aggressive Large B-Cell Lymphomas: A Retrospective Single-Center Study

**DOI:** 10.3390/diagnostics16203349

**Published:** 2026-10-14

**Authors:** Serhat Kaymaz, Selçuk Cin, Tuğrul Elverdi, Suat Hilal Akı, Ahu Senem Demiröz

**Affiliations:** 1 Department of Pathology, Cerrahpaşa Faculty of Medicine, Istanbul University-Cerrahpaşa, 34098 Istanbul, Turkey; 2 Department of Hematology, Cerrahpaşa Faculty of Medicine, Istanbul University-Cerrahpaşa, 34098 Istanbul, Turkey

**Keywords:** PD-L1, large B-cell lymphoma, primary mediastinal large B-cell lymphoma, mediastinal gray zone lymphoma, diffuse large B-cell lymphoma, immunohistochemistry, immune checkpoint

## Abstract

**Background:** Programmed death-ligand 1 (PD-L1) contributes to immune escape and is a therapeutically relevant target in aggressive B-cell lymphomas. However, its distribution and clinicopathologic significance across primary mediastinal aggressive large B-cell lymphomas remain incompletely defined. **Methods:** We retrospectively reviewed 53 cases diagnosed between 2010 and 2025, including diffuse large B-cell lymphoma (DLBCL), primary mediastinal large B-cell lymphoma (PMBCL), mediastinal gray zone lymphoma (MGZL), and T-cell/histiocyte-rich large B-cell lymphoma (THRLBCL). PD-L1 immunohistochemistry (clone 22C3) was performed on formalin-fixed, paraffin-embedded tissue. Tumor cells and intratumoral inflammatory cells were assessed together. Membranous staining was scored as 0, 1+, 2+, or 3+, and the proportion of cells in each category was recorded. **Results:** PD-L1 staining was present in 40/53 cases (75.5%; 95% CI, 61.7–86.2%). Mean PD-L1-positive percentages were similar in DLBCL, PMBCL, and MGZL (38.81%, 31.67%, and 43.89%, respectively; *p* = 0.726), and no tested cutoff separated the diagnostic groups. In contrast, MGZL showed a higher mean 3+ component (31.11%) and more cases with 3+ staining in at least 50% of evaluated cells (4/9, 44.4%; 95% CI, 13.7–78.8%) than DLBCL (1/21, 4.8%; 95% CI, 0.1–23.8%) or PMBCL (1/21, 4.8%; 95% CI, 0.1–23.8%); the overall comparison remained significant after Bonferroni correction (unadjusted *p* = 0.012; adjusted *p* = 0.049). PD-L1 extent was not associated with age, tumor size, LDH, beta-2 microglobulin, Ann Arbor stage, SUVmax, treatment response, or overall survival. **Conclusions:** PD-L1 expression is common in mediastinal aggressive large B-cell lymphomas. MGZL showed an exploratory strong-staining pattern that requires independent validation. No prognostic association was detected in this cohort.

## 1. Introduction

Mediastinal aggressive large B-cell lymphomas form a small but clinically important group of tumors in which anatomic site, morphology, immunophenotype, and molecular background must be interpreted together. The group includes primary mediastinal large B-cell lymphoma (PMBCL), mediastinal gray zone lymphoma (MGZL), diffuse large B-cell lymphoma (DLBCL) presenting as primary mediastinal disease, and, less commonly, T-cell/histiocyte-rich large B-cell lymphoma (THRLBCL) [1,2,3,4,5]. Although these entities share a mediastinal presentation and aggressive behavior, they differ in age distribution, sex predominance, tumor architecture, composition of the microenvironment, and response to treatment. Correct classification is therefore relevant not only for diagnosis but also for selection of systemic therapy and interpretation of emerging biomarkers.

PMBCL is a distinct large B-cell lymphoma thought to arise from thymic medullary B cells. It usually affects young adults, more often women, and commonly presents as a rapidly enlarging anterior mediastinal mass that may be bulky and may extend to adjacent thoracic structures [2,5,6]. Histologically, the tumor is composed of medium-to-large atypical B cells, often with pale cytoplasm, separated by fine fibrous bands. The neoplastic cells retain a B-cell program but frequently show features that overlap with classical Hodgkin lymphoma (CHL), including CD30 expression and recurrent activation of the JAK-STAT and NF-κB pathways [2,5,6,7]. These similarities are especially relevant to immune escape because PMBCL and CHL frequently harbor alterations of chromosome 9p24.1.

MGZL lies at the interface between PMBCL and CHL and is defined by discordance between morphology and immunophenotype. Some cases resemble CHL morphologically but retain a strong B-cell program, whereas others resemble PMBCL but show reduced B-cell marker expression and stronger CD30 and/or CD15 expression [3,5,8,9,10,11,12,13]. The diagnosis can be difficult in small biopsies because the characteristic heterogeneity may not be represented. MGZL is also clinically relevant because it is rare, often affects young adults, has a male predominance in several series, and generally has less favorable outcomes than PMBCL. The absence of a universally accepted treatment approach further increases interest in biologically directed therapies [3,9,10].

DLBCL is the most common non-Hodgkin lymphoma and is itself biologically heterogeneous [1,14]. Mediastinal DLBCL must be distinguished from PMBCL by integrating clinical presentation, histomorphology, and immunophenotype. THRLBCL represents a different pattern of large B-cell lymphoma, in which scattered neoplastic B cells constitute less than 10% of the cellular population and are embedded in a prominent background of reactive T cells and histiocytes [4,15,16]. This microenvironment-rich architecture makes THRLBCL particularly relevant to studies of immune regulation, but it also complicates attribution of biomarker expression to malignant versus non-neoplastic cells.

Programmed cell death protein 1 (PD-1) is an inhibitory receptor expressed mainly on activated T cells and also on subsets of B cells and natural killer cells. Binding of PD-1 to programmed death-ligand 1 (PD-L1) suppresses T-cell proliferation, cytokine production, and cytotoxic activity, thereby limiting excessive immune responses under physiologic conditions [17,18,19]. Tumors can exploit the same pathway by expressing PD-L1 on neoplastic cells or by inducing PD-L1 expression in macrophages and other cells of the tumor microenvironment. Sustained signaling through this axis contributes to T-cell dysfunction or exhaustion and permits immune evasion [17,18,19,20,21]. Antibodies that block PD-1 or PD-L1 can restore antitumor immune activity and have shown marked efficacy in selected lymphomas, most clearly in CHL and in relapsed or refractory PMBCL [20,21,22].

The mechanisms producing PD-L1 expression are not identical across large B-cell lymphomas. In PMBCL, recurrent copy-number gains, amplification, or rearrangement of 9p24.1 increase expression of PD-L1 and PD-L2; co-amplification of JAK2 can further enhance checkpoint-ligand expression through JAK-STAT signaling [5,6,7]. MGZL shares many of these molecular features with PMBCL and CHL, including frequent 9p24.1 abnormalities [8,9,10,11,12,13,23]. In DLBCL, PD-L1 expression is more variable and may be associated with activated B-cell-like biology, NF-κB activation, viral signaling in Epstein-Barr virus-associated cases, or microenvironmental induction [24,25,26,27,28]. In THRLBCL, PD-1 pathway activity appears to involve both the sparse tumor cells and the abundant surrounding macrophages and T cells, consistent with an adaptive immune-resistance pattern [15,16].

Despite its biologic and therapeutic relevance, PD-L1 immunohistochemistry has not been standardized in lymphoma. Solid tumors are assessed using assay-specific systems such as tumor proportion score, combined positive score, or immune-cell score, with predefined antibody clones and treatment-linked cutoffs [29]. In lymphomas, published studies differ in antibody clone, staining platform, cell population evaluated, and positivity threshold. Some analyses score only tumor cells, whereas others separately or jointly assess macrophages, lymphocytes, and other intratumoral immune cells [24,25,26,27,29]. This variability is particularly consequential in MGZL and THRLBCL, where neoplastic cells may be sparse or difficult to distinguish from the inflammatory background. Moreover, most studies reduce PD-L1 to a positive/negative result at a single cutoff, even though staining intensity and intratumoral heterogeneity may contain additional information.

The present study was designed to characterize PD-L1 expression in a single-center series of primary mediastinal aggressive large B-cell lymphomas using a combined assessment of tumor cells and intratumoral inflammatory cells. Both the overall proportion of PD-L1-positive cells and the distribution of weak, moderate, and strong membranous staining were recorded. We then examined whether PD-L1 expression differed among diagnostic categories and whether it was associated with age, tumor size, serum laboratory variables, Ann Arbor stage, metabolic activity on positron emission tomography, treatment response, or overall survival.

## 2. Materials and Methods

### 2.1. Study Design and Case Selection

This retrospective single-center study was conducted in the Department of Pathology, Cerrahpaşa Faculty of Medicine, Istanbul University-Cerrahpaşa, using archived material from cases diagnosed between 2010 and 2025. The pathology archive and institutional digital records were searched for non-Hodgkin large B-cell lymphomas presenting in the mediastinum. Cases with secondary mediastinal involvement as part of systemic disease were excluded; only tumors considered primary to the mediastinal presentation were retained.

Eighty patients were initially identified. All available hematoxylin and eosin (H&E)-stained slides and prior immunohistochemical studies were reviewed, and the corresponding paraffin blocks were retrieved. Twenty-seven patients were excluded because the residual tumor tissue was insufficient, the block was exhausted, or the tumor-containing block had previously been released from the archive. Case-level demographic and clinical data were not available for these 27 excluded patients; therefore, a formal comparison with the included cohort could not be performed. The final cohort consisted of 53 patients: 21 DLBCL, 21 PMBCL, 9 MGZL, and 2 THRLBCL. For each case, the H&E slides were re-examined, and one formalin-fixed, paraffin-embedded (FFPE) block containing the largest and best-preserved representative tumor area was selected. Diagnostic classification was based on the original pathologic findings together with a review of morphology and the available immunophenotype, according to the fifth edition of the World Health Organization Classification of Haematolymphoid Tumours (WHO-HAEM5) [5]. Cell-of-origin classification of DLBCL was assessed using the Hans immunohistochemical algorithm based on CD10, BCL6, and MUM1 [27].

The study was conducted in accordance with the Declaration of Helsinki and was approved by the Non-Interventional Clinical Research Ethics Committee of Istanbul University-Cerrahpaşa Rectorate (decision no. 2025/409; approval date: 11 June 2025).

### 2.2. Immunohistochemistry

Sections 2 μm thick were cut from the selected FFPE blocks and mounted on positively charged slides. The slides were incubated in a 70 °C oven for approximately 20–30 min before automated staining. Deparaffinization was performed on the instrument with EZ Prep solution (Ventana Medical Systems, Inc., Tucson, AZ, USA) solution. Antigen retrieval was carried out with CC1 solution (EDTA, pH 9; Ventana Medical Systems, Inc., Tucson, AZ, USA) at 97 °C for 120 min. Reaction Buffer and LCS (both from Ventana Medical Systems, Inc., Tucson, AZ, USA) were used during automated staining.

PD-L1 immunohistochemistry was performed with the monoclonal antibody clone 22C3 (Agilent (Santa Clara, CA, USA)/Dako (Carpinteria, CA, USA), M365329-2) at a 1:50 dilution on a BenchMark ULTRA platform (Ventana Medical Systems, Inc., Tucson, AZ, USA). The primary antibody was incubated for 120 min. Detection was performed using the ultraView Universal DAB Detection Kit (Ventana Medical Systems, Inc., Tucson, AZ, USA) and ultraview Universal HRP secondary antibody was applied for 8 min, followed by 3,3′-diaminobenzidine development for 8 min and copper enhancement for 4 min. Counterstaining and bluing were performed on the instrument using hematoxylin and Bluing Reagent, respectively (both from Ventana Medical Systems, Inc., Tucson, AZ, USA). Tonsil tissue was used as an external control; weak membranous staining in lymphoid and epithelial compartments was accepted as an adequate positive control.

### 2.3. PD-L1 Evaluation

For each case, the evaluable tumor region was first delineated on the corresponding H&E slide. Necrosis, crush or cautery artifact, and non-neoplastic tissue outside the tumor were excluded. PD-L1-stained slides were assessed in a joint session by three observers, comprising two hematopathologists and one pathology resident, using light microscopy. Partial or complete membranous staining was considered positive. Purely cytoplasmic, granular, or otherwise nonspecific staining was not counted.

Tumor cells and inflammatory cells located within the defined tumor area, including intratumoral lymphocytes and histiocytes, were assessed together. Inflammatory cells outside tumor islands or outside the selected tumor region were excluded. This combined approach was chosen because the PD-1/PD-L1 axis can operate through both malignant cells and the tumor microenvironment and because reliable lineage assignment is difficult in some MGZL and THRLBCL specimens when only a single PD-L1 stain is available [24,26,27,29].

**Figure 1:**
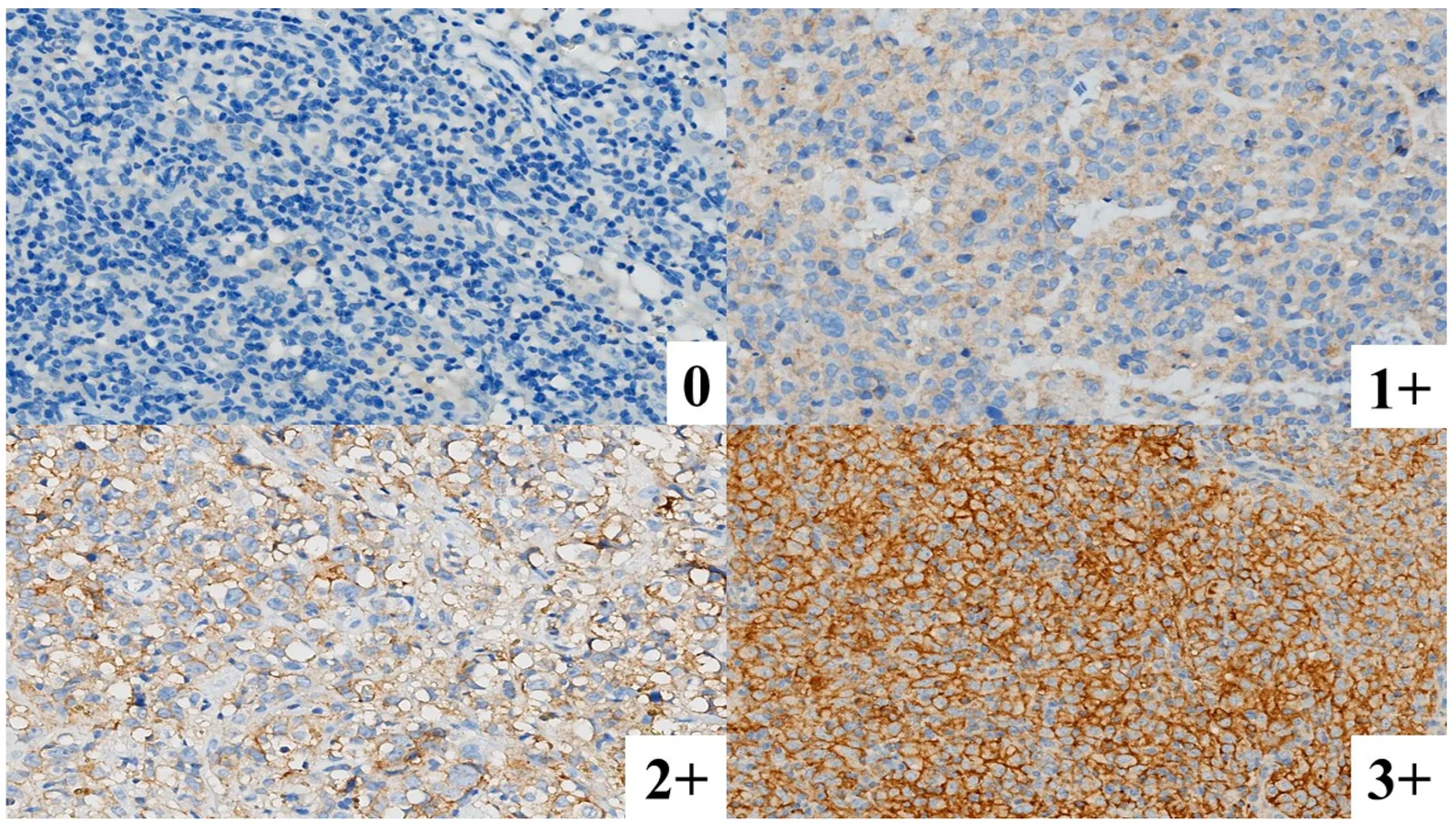
Representative examples of PD-L1 immunohistochemical membranous staining intensities scored as 0, 1+, 2+, and 3+. All panels were photographed at 20× objective magnification.

Staining intensity was categorized as 0 (no staining), 1+ (weak), 2+ (moderate), or 3+ (strong), consistent with semiquantitative intensity grading used in prior lymphoma studies [23,27,28]. The entire tumor area was surveyed, and more than one representative field was considered when staining was heterogeneous. For each intensity category, the proportion of all evaluated tumor and intratumoral inflammatory cells was estimated as a percentage. The four percentages were required to sum to 100% in every case. Overall PD-L1 extent was calculated as the combined proportion of cells showing 1+, 2+, or 3+ membranous staining. The three observers reviewed the slides simultaneously and assigned a consensus value at the time of evaluation; observer-specific pre-consensus scores were not generated or recorded. Representative staining patterns are shown in Figure 1.

### 2.4. Clinical Data Collection and Definitions

Clinical data were collected retrospectively from the hospital digital information system and the archive of the Department of Hematology. The index diagnosis date, treatment initiation and completion dates, treatment regimens, follow-up notes, discharge summaries, and last known clinical status were recorded. For seven patients who were not followed at the institution and for whom the available records were insufficient, the patient or a relative was contacted to establish the last known status and obtain missing treatment information. All variables were entered into a standardized data collection form.

Baseline laboratory and imaging variables included serum lactate dehydrogenase (LDH), serum beta-2 microglobulin, largest tumor diameter, Ann Arbor stage, and maximum standardized uptake value (SUVmax) on fluorodeoxyglucose positron emission tomography/computed tomography. Treatment duration was calculated from the first day of systemic therapy to the final treatment date; for patients still receiving treatment, the last follow-up date was used. Hematopoietic stem cell transplantation and radiotherapy were recorded when applicable. Last known status was categorized as alive and disease-free, alive under treatment or observation, relapsed/refractory disease, or deceased. For the exploratory response analysis, the treating records were used to classify patients as having a treatment response or no treatment response. Unavailable values were coded as missing and were excluded only from the relevant analysis.

### 2.5. Statistical Analysis

Statistical analyses were performed using IBM SPSS Statistics, version 32.0 (IBM Corp., Armonk, NY, USA). Continuous variables were summarized using mean, median, interquartile range (IQR), and range as appropriate, and categorical variables were summarized as counts and percentages. Normality was assessed with the Shapiro–Wilk test. For independent-group comparisons, the Mann–Whitney U test was used for two groups and the Kruskal–Wallis test for more than two groups. When the Kruskal–Wallis test was significant, Dunn post hoc comparisons with Bonferroni adjustment were performed. Associations between continuous variables were evaluated using Pearson’s correlation coefficient. Exact binomial 95% confidence intervals (CIs) were calculated for the principal proportions.

Categorical variables were compared using Fisher’s exact test; for tables larger than 2 × 2, the Fisher–Freeman–Halton extension was used. Cramer’s V was calculated as an effect-size measure when appropriate. Because no universally accepted PD-L1 cutoff exists for lymphoma, PD-L1 was analyzed as a continuous percentage and at thresholds of 1%, 5%, 10%, 20%, 30%, 40%, 50%, 60%, and 70%. These cutoff analyses were exploratory; Bonferroni correction was applied separately across the six reported diagnostic-group cutoff comparisons. The primary outcome was the comparison of overall PD-L1-positive percentage among DLBCL, PMBCL, and MGZL. Secondary outcomes were the between-group comparisons of the proportions of cells showing 1+, 2+, and 3+ staining and the prespecified endpoint of 3+ staining in at least 50% of evaluated cells. The 50% threshold was specified before analysis. Bonferroni correction was applied across these four secondary intensity outcomes. THRLBCL was not included in the secondary intensity comparisons and was described separately. The original cutoff analyses, in which THRLBCL was combined with DLBCL because of the subgroup size, were retained as exploratory analyses and are identified explicitly.

Overall survival was defined as the interval from diagnosis to death. Survival probabilities were estimated by the Kaplan–Meier method, and curves were compared with the log-rank test. Within diagnostic groups, the relationship between PD-L1 percentage and overall survival was explored using Cox proportional hazards regression. These diagnosis-specific Cox analyses were considered exploratory because of the low number of events. One patient without adequate follow-up duration was excluded from time-to-event analyses. A two-sided *p* value < 0.05 was considered statistically significant.

## 3. Results

### 3.1. Clinicopathologic Characteristics

A total of 53 patients with mediastinal aggressive large B-cell lymphomas were included. The cohort comprised 21 DLBCL, 21 PMBCL, 9 MGZL, and 2 THRLBCL cases. The median age at diagnosis was 44 years (IQR, 28–55 years; range, 14–93 years), with visible clustering in the third and sixth decades. There were 28 female patients (52.8%) and 25 male patients (47.2%). The DLBCL group included 13 women and 8 men, and the PMBCL group included 12 women and 9 men. MGZL showed a male predominance, with 6 men and 3 women, while both THRLBCL patients were men.

At last follow-up, 31 patients (58.5%) were alive and disease-free, 2 (3.8%) were alive under active treatment, 3 (5.7%) had relapsed or refractory disease, and 17 (32.1%) had died. By diagnosis, 15/21 DLBCL patients, 10/21 PMBCL patients, 4/9 MGZL patients, and both THRLBCL patients were alive and disease-free. Deaths occurred in 5 DLBCL, 8 PMBCL, 4 MGZL, and 0 THRLBCL patients; because one deceased PMBCL patient lacked a recorded follow-up duration, the time-to-event analyses included 5, 7, 4, and 0 events, respectively. The estimated 5-year overall survival for the entire cohort was 67.3%; the corresponding diagnosis-specific estimates were 76.2% for DLBCL, 58.6% for PMBCL, 55.6% for MGZL, and 100% for THRLBCL. The THRLBCL estimate should be interpreted only descriptively because it was based on two patients.

Ten patients underwent hematopoietic stem cell transplantation. The mean treatment duration was 11.7 months. R-CHOP was the most frequently administered systemic regimen, and radiotherapy was added in five patients. Treatment approaches were heterogeneous because the cohort covered a 15-year period and included several diagnostic categories.

### 3.2. Associations Between PD-L1 Expression and Clinical Parameters

PD-L1 staining percentage did not differ significantly between patients aged <60 years and those aged ≥60 years (*p* = 0.16). The median PD-L1 percentages in these groups were 35% and 10%, respectively, but the distributions overlapped substantially. PD-L1 was also not associated with serum LDH (<280 vs. ≥280 U/L; *p* = 0.50), serum beta-2 microglobulin (<2200 vs. ≥2200 ng/mL; *p* = 0.98), tumor size (<10 vs. ≥10 cm; *p* = 0.36), or Ann Arbor stage (*p* = 0.55) (Table 1).

The mean serum LDH value in the cohort was 466 U/L. Mean values by diagnosis were 538.8 U/L in PMBCL, 462.5 U/L in DLBCL, and 348.3 U/L in MGZL. The median PD-L1 percentage was 40% in patients with LDH below 280 U/L and 30% in those with elevated LDH. The mean serum beta-2 microglobulin value was 3607 ng/mL; median PD-L1 percentages were 55% in patients below 2200 ng/mL and 30% in those at or above this threshold. Neither comparison reached statistical significance.

Tumor size differed significantly among diagnostic groups (Kruskal–Wallis *p* < 0.001). The median largest diameter was 10 cm in PMBCL, 5 cm in DLBCL, and 2.7 cm in MGZL. Post hoc analysis showed that PMBCL tumors were significantly larger than the other groups (Dunn–Bonferroni *p* = 0.0075). SUVmax also differed among diagnoses (*p* = 0.001). Mean SUVmax was 24.31 in PMBCL, 20.98 in DLBCL, and 9.85 in MGZL; MGZL values were lower than those of PMBCL and DLBCL in post hoc comparisons. Despite these diagnosis-related differences in anatomic and metabolic tumor characteristics, PD-L1 percentage was not correlated with SUVmax (*p* = 0.922) and was not associated with tumor size.

### 3.3. PD-L1 Expression Across Diagnostic Groups

**Figure 2:**
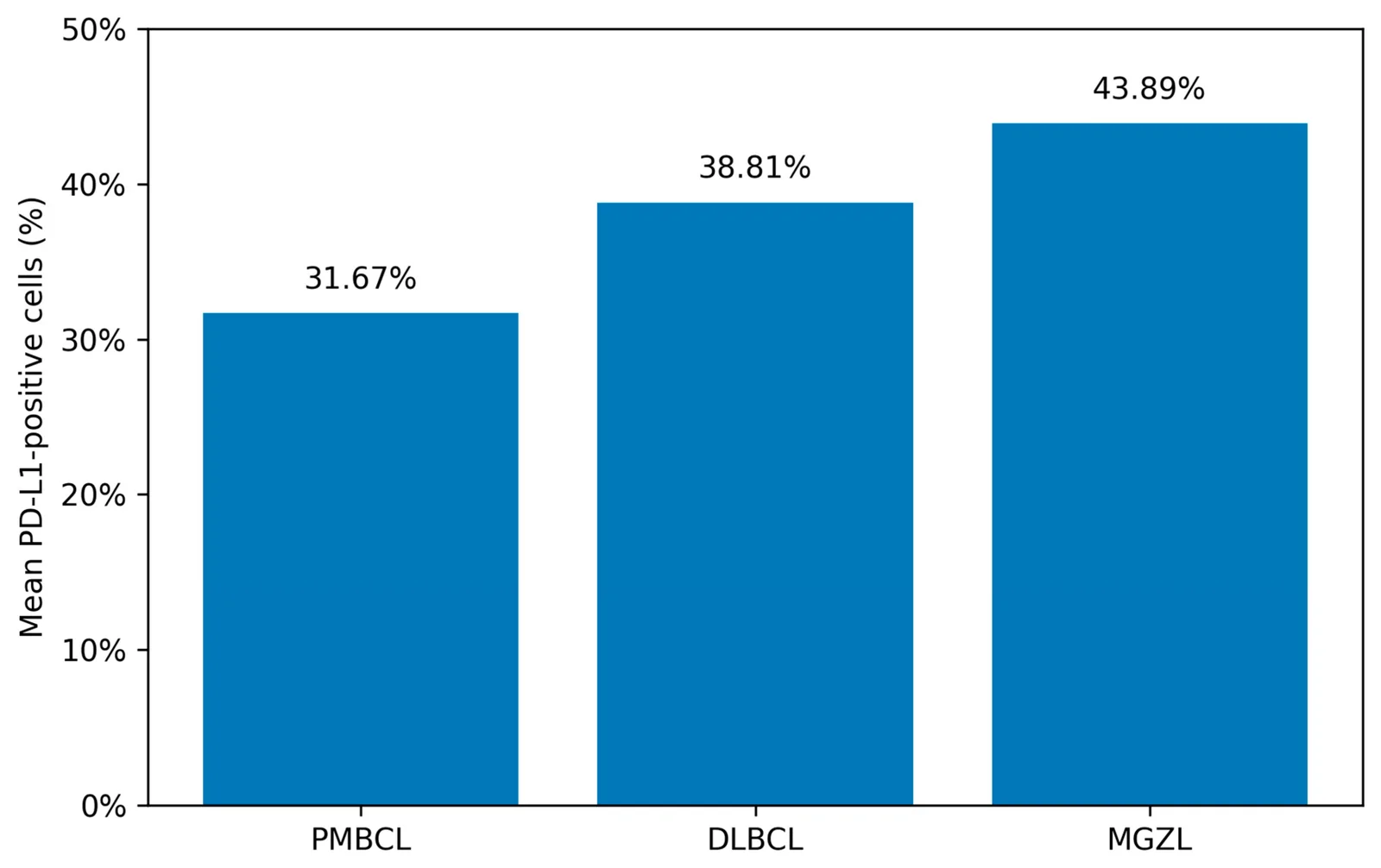
Mean overall PD-L1-positive cell percentage according to diagnostic category.

Overall, 40/53 patients (75.5%; 95% CI, 61.7–86.2%) showed PD-L1 staining, whereas 13/53 (24.5%) showed no membranous staining. Mean overall PD-L1-positive cell percentages were 38.81% for DLBCL, 31.67% for PMBCL, and 43.89% for MGZL. These distributions were similar across diagnostic groups (Kruskal–Wallis *p* = 0.726) (Figure 2).

Because no universal cutoff exists for PD-L1 positivity in lymphoma, multiple thresholds were examined as exploratory analyses. At cutoffs of ≥5%, ≥10%, ≥20%, ≥30%, ≥50%, and ≥70%, positivity rates did not differ significantly among the diagnostic groups (unadjusted *p* = 0.531, 0.618, 0.809, 0.708, 0.494, and 0.572, respectively; Bonferroni-adjusted *p* = 1.000 for all six comparisons). At the ≥5% threshold, 15/21 PMBCL cases (71.4%), 19/23 cases in the DLBCL/THRLBCL-combined group (82.6%), and 6/9 MGZL cases (66.7%) were positive. These original cutoff analyses were retained as exploratory and were not used for entity-specific inference because the two THRLBCL cases were included in the combined DLBCL/THRLBCL group.

Within the DLBCL group, 14 cases were classified as non-GCB, 2 as germinal-center B-cell-like, and 5 as unclassifiable by the available immunohistochemical algorithm. Median PD-L1 percentages were 36%, 90%, and 32%, respectively. The difference approached but did not reach statistical significance (*p* = 0.06). The apparently high value in the germinal-center group was based on only two cases and is reported descriptively; no inference regarding GCB-like DLBCL can be made.

### 3.4. PD-L1 Staining Intensity Patterns

**Figure 3:**
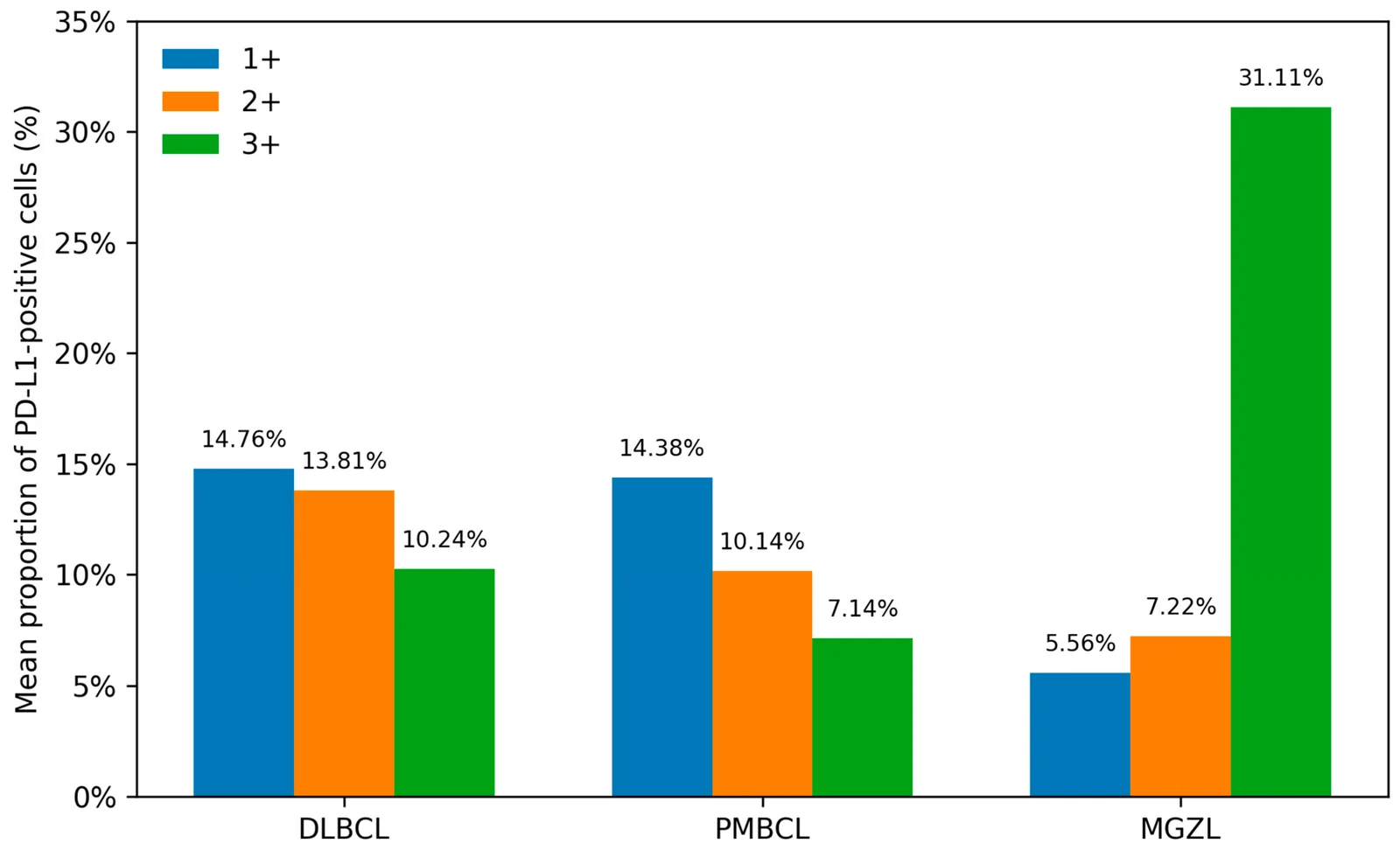
Mean proportions of cells showing 1+, 2+, and 3+ PD-L1 membranous staining across diagnostic groups.

The intensity distribution differed more clearly than the overall PD-L1-positive percentage. In DLBCL, after excluding the two THRLBCL cases, the mean proportions of cells showing 1+, 2+, and 3+ staining were 14.76%, 13.81%, and 10.24%, respectively. The corresponding values were 14.38%, 10.14%, and 7.14% in PMBCL and 5.56%, 7.22%, and 31.11% in MGZL (Figure 3). Therefore, the higher overall PD-L1 value in MGZL was driven mainly by strong membranous staining rather than by a larger weakly positive compartment.

Cases in which at least 50% of evaluated cells showed 3+ staining were uncommon in DLBCL and PMBCL but frequent in MGZL. The proportions were 1/21 (4.8%; 95% CI, 0.1–23.8%), 1/21 (4.8%; 95% CI, 0.1–23.8%), and 4/9 (44.4%; 95% CI, 13.7–78.8%), respectively. The overall difference remained significant after Bonferroni correction across the four secondary intensity outcomes (Fisher–Freeman–Halton unadjusted *p* = 0.012; adjusted *p* = 0.049; Cramer’s V = 0.47). No pairwise comparison remained significant after Bonferroni correction (adjusted *p* = 0.058 for MGZL versus DLBCL and for MGZL versus PMBCL; adjusted *p* = 1.000 for DLBCL versus PMBCL). Case-level MGZL intensity distributions are provided in Supplementary Table S1.

### 3.5. PD-L1 Expression, Treatment Response, and Survival

Across PD-L1 positivity thresholds from ≥1% to ≥70%, PD-L1 status was not associated with vital status (alive versus deceased; all *p* > 0.05). The mean observed overall survival time was 66.51 months. Kaplan–Meier comparisons at cutoffs of 5%, 10%, 20%, 30%, 50%, and 70% showed no significant survival difference between PD-L1-positive and PD-L1-negative groups (log-rank p = 0.82, 0.61, 0.67, 0.54, 0.41, and 0.52, respectively). In diagnosis-specific Cox analyses, PD-L1 percentage was not significantly related to overall survival in DLBCL (*p* = 0.10; 5 events), PMBCL (*p* = 0.64; 7 events), or MGZL (*p* = 0.62; 4 events). Given these event counts, the Cox results are exploratory.

A treatment response was documented in 42/53 patients (79%; 95% CI, 65.9–89.2%), whereas 11/53 patients (21%) were classified as having no response. The mean PD-L1 percentage was 40% among responders and 32% among nonresponders. This difference was not statistically significant (Mann–Whitney U test, *p* = 0.36).

## 4. Discussion

This study evaluated PD-L1 in a diagnostically heterogeneous but anatomically defined cohort of mediastinal aggressive large B-cell lymphomas. Three findings are central. First, PD-L1 expression was common, occurring in 75.5% of cases when any membranous staining was accepted. Second, overall PD-L1-positive percentages were similar among DLBCL, PMBCL, and MGZL. Third, MGZL showed an exploratory intensity pattern: strong 3+ staining accounted for a larger proportion of evaluated cells, and four of nine MGZL cases had 3+ staining in at least half of the tumor-area cellular population. The global comparison remained significant after Bonferroni correction, although the confidence interval for the MGZL proportion was wide and adjusted pairwise comparisons were not significant. No association was detected between PD-L1 extent and the clinical variables examined, treatment response, or overall survival; these negative findings should be interpreted in light of the sample size and low event counts.

The combined scoring method used in this study requires specific consideration. PD-L1 may be expressed by malignant B cells, macrophages, histiocytes, and lymphocytes within the same biopsy [24,26,27,29]. Separating these compartments is feasible when morphology is clear or when lineage-specific double staining is available, but it becomes difficult in fibrotic PMBCL, pleomorphic MGZL, and microenvironment-rich THRLBCL. We therefore assessed tumor cells and intratumoral inflammatory cells together and recorded both extent and intensity. Tumor proportion score (TPS) uses PD-L1-positive tumor cells as the numerator and viable tumor cells as the denominator, whereas combined positive score (CPS) includes PD-L1-positive tumor cells and selected immune cells in the numerator but retains viable tumor cells as the denominator [29]. Our method differs from both: the numerator and denominator included tumor cells and intratumoral inflammatory cells, and the distribution was recorded separately at 0, 1+, 2+, and 3+ intensity. It is therefore neither TPS nor CPS and is not directly equivalent to lymphoma studies that score neoplastic and immune compartments separately. It instead describes the total PD-L1-positive cellular compartment within the tumor region. For a study focused on immune escape in mediastinal lymphomas, this captures a biologically relevant feature of the microenvironment, but it merges biologically distinct populations and limits direct comparison with studies that score malignant cells alone. Future studies should separate compartments using lineage-specific double labeling, such as PD-L1/PAX5, or validated digital image analysis.

The baseline clinicopathologic findings were consistent with several established features of these diseases. PMBCL showed a slight female predominance and had the largest median tumor diameter, reaching 10 cm, which is compatible with its usual presentation as a bulky anterior mediastinal mass [2,6]. MGZL showed a male predominance, in keeping with larger published series [3,9]. Tumor size and SUVmax differed among diagnoses, with PMBCL showing the largest masses and MGZL the lowest mean SUVmax. These differences confirm that the diagnostic groups were not clinically interchangeable. However, neither tumor size nor SUVmax was associated with PD-L1 percentage. PD-L1 expression in this setting may therefore reflect the tumor’s molecular identity and immune interactions more than simple tumor burden or metabolic activity.

PMBCL is one of the non-Hodgkin lymphomas in which the biologic basis for checkpoint-ligand expression is best established. Gains and amplification of 9p24.1 involve PD-L1, PD-L2, and JAK2 and can increase ligand expression through both gene dosage and JAK-STAT activation [5,6,7,30]. Published PD-L1 positivity rates in PMBCL vary widely because of differences in clone, threshold, and whether tumor or immune cells are counted [24]. The mean PD-L1 percentage of 31.67% in our PMBCL cases falls within this broad published range. Nevertheless, only a small fraction of PMBCL cases showed 3+ staining in at least half of the evaluated cells. This apparently modest strong-staining burden may reflect the combined denominator used in our scoring method, intratumoral heterogeneity, or technical differences from studies that focus specifically on neoplastic cells. It does not negate the therapeutic relevance of the PD-1 pathway in relapsed or refractory PMBCL [20,21,22].

MGZL showed the most pronounced 3+ component in the intensity analysis. The mean overall PD-L1 percentage was only moderately higher than in PMBCL and DLBCL, but the composition of that positivity was different: the mean 3+ component was 31.11%, and four of nine cases showed 3+ staining in at least 50% of evaluated cells. The global comparison remained significant after Bonferroni correction, but the 95% CI for the MGZL proportion was wide, and adjusted pairwise comparisons were not significant. This finding is concordant with the close molecular relationship among MGZL, PMBCL, and CHL and with reports of frequent 9p24.1 alterations and strong PD-L1 staining in MGZL [8,9,10,11,12,13,23]. Pelland et al. reported strong PD-L1 expression in their small MGZL series [23]. Because MGZL is rare and diagnostically difficult, this result should not be used as an isolated diagnostic criterion. It is better regarded as an exploratory supportive biologic pattern that warrants confirmation in larger, independently reviewed cohorts.

The therapeutic implications of our findings in MGZL remain to be established. Conventional treatment is not standardized, and outcomes have often been less favorable than those of PMBCL [3,9,10]. Strong PD-L1 staining is compatible with the activity of the PD-1 axis and with the reported activity of checkpoint blockade in relapsed or refractory B-cell lymphomas [21,22]. However, no patient in this cohort received a PD-1 or PD-L1 inhibitor. The present staining results therefore cannot be used to infer treatment response or to select patients for checkpoint blockade. Prospective or multicenter retrospective studies linking a standardized lineage-specific PD-L1 score, 9p24.1 status, and actual checkpoint-inhibitor outcomes would be needed before this pattern could be used predictively.

PD-L1 expression in DLBCL is more heterogeneous. Prior studies have reported associations with activated B-cell-like or non-germinal-center phenotype, adverse clinicopathologic features, and inferior survival, but other cohorts have not reproduced a consistent prognostic effect [24,25,26,27,28]. Our mediastinal DLBCL cases showed frequent PD-L1 positivity, with a mean of 38.81%, yet neither treatment response nor overall survival was related to PD-L1. Fourteen of 21 DLBCL cases were non-GCB, which may partly explain the relatively high expression in this group. The germinal-center subgroup had a mean PD-L1 percentage of 90%, but it contained only two cases. This descriptive value is too unstable to support an inference regarding GCB-like DLBCL. The result illustrates how unstable subgroup estimates can become in small series.

THRLBCL is especially dependent on its non-neoplastic cellular background. Spatial and transcriptional studies have described a tolerogenic microenvironment in which PD-1-positive T cells and PD-L1-positive macrophages surround sparse malignant B cells [15,16]. Both THRLBCL cases in our cohort showed PD-L1 staining, but neither met the prespecified ≥50% 3+ endpoint. They were excluded from the secondary intensity comparisons and are reported only descriptively. No entity-specific conclusion for THRLBCL can be drawn from two cases.

No significant relationship was found between PD-L1 extent and age, LDH, beta-2 microglobulin, tumor size, Ann Arbor stage, or SUVmax. The literature on these associations is inconsistent, particularly in DLBCL [25,26,27]. In our cohort, patients younger than 60 years had a higher PD-L1 percentage than older patients, and early-stage cases included some high-expression tumors, but neither pattern was statistically significant. The concentration of PMBCL and MGZL among younger patients and the tendency of PMBCL to remain anatomically localized despite a large mediastinal mass may contribute to these observations. A recent DLBCL series reported an association between PD-L1 expression and higher SUVmax, but our mediastinal cohort did not reproduce that relationship [31]. The absence of association with conventional burden-related variables supports the view that PD-L1 is not simply a surrogate for advanced stage or larger tumor volume.

PD-L1 also lacked a significant association with overall survival at every tested cutoff. Studies reporting adverse prognostic effects have often assessed tumor-cell PD-L1 with a single predefined threshold, whereas our score included the intratumoral immune compartment [26]. PD-L1 positivity in macrophages or lymphocytes may indicate an active host response as well as an immunosuppressive pathway; these opposing biologic meanings can weaken a simple survival association. In addition, the cohort contained several entities with different natural histories and treatment approaches. The diagnosis-specific Cox analyses included only 5 DLBCL, 7 PMBCL, and 4 MGZL events. The present results therefore do not establish the absence of prognostic value. The same considerations apply to treatment response. Most patients received conventional immunochemotherapy rather than checkpoint blockade, consistent with standard treatment approaches for aggressive large B-cell lymphoma [32], and PD-L1 is not expected to function as a direct marker of sensitivity to cytotoxic agents. Because no patient received a PD-1/PD-L1 inhibitor, the treatment-response analysis cannot be extrapolated to predict response to checkpoint blockade.

The study has several strengths. All cases arose in the mediastinal setting; archived slides and blocks were re-reviewed; one representative tumor-rich block was selected for each patient; and staining was evaluated by three observers with consensus resolution. The analysis did not rely on a single arbitrary PD-L1 cutoff; multiple thresholds were tested, and intensity-specific proportions were recorded. This allowed identification of the strong-staining MGZL pattern, which would have been less visible in a binary positive/negative analysis.

The limitations are equally important. The retrospective single-center design and the rarity of MGZL and THRLBCL restricted subgroup size and statistical power. Observer-level reproducibility could not be assessed because the three observers evaluated the slides simultaneously and only consensus values were recorded. One block cannot fully represent spatial heterogeneity within a large mediastinal mass. The combined score could not distinguish PD-L1 expression in tumor cells from that in macrophages or lymphocytes, and no PD-L1/PAX5 double staining, digital image analysis, 9p24.1 testing, or other integrated molecular assessment was available. Twenty-seven of the 80 initially identified patients were excluded because adequate residual archival tissue was unavailable. As no case-level demographic or clinical data were available for these excluded patients, selection bias could not be quantified; the final cohort may preferentially represent larger biopsies, better-preserved material, or cases with more residual tissue. The original exploratory cutoff analyses combined the two THRLBCL cases with DLBCL, which may have introduced biological heterogeneity. Diagnosis-specific Cox analyses were based on few events and were underpowered. Treatment was heterogeneous over the 2010–2025 period, and no patient received a checkpoint inhibitor, preventing direct evaluation of predictive value. Accordingly, the findings apply to a predominantly conventional immunochemotherapy setting and cannot be extrapolated to predict response to PD-1/PD-L1 blockade. Future studies should use multicenter cohorts, central diagnostic review, independent observer scoring, standardized assay conditions, separate tumor and immune-cell scoring, and molecular correlation. For MGZL in particular, staining intensity should be retained as an analytic variable rather than reducing PD-L1 to a single cutoff.

## 5. Conclusions

PD-L1 expression was frequent in primary mediastinal aggressive large B-cell lymphomas, but overall staining extent did not differ significantly among DLBCL, PMBCL, and MGZL. No association with the clinicopathologic variables, treatment response, or overall survival was detected in the exploratory analyses. These negative findings are limited by the combined tumor/microenvironment score, small subgroups, treatment heterogeneity, and few events; they do not establish that PD-L1 lacks prognostic value. Because the cohort was treated predominantly with conventional immunochemotherapy and included no checkpoint-inhibitor recipients, no predictive conclusion regarding PD-1/PD-L1 blockade can be made.

MGZL showed an exploratory enrichment for strong 3+ staining, with four of nine cases meeting the prespecified ≥50% 3+ endpoint. The global comparison remained significant after Bonferroni correction, but the confidence interval was wide, and adjusted pairwise comparisons were not significant. This observation is compatible with the biologic relevance of the PD-1/PD-L1 axis in MGZL but requires independent validation in larger, centrally reviewed cohorts using lineage-specific scoring and 9p24.1 correlation. It should not be used as a diagnostic or predictive criterion.

## Figures and Tables

**Table 1:** Associations between PD-L1 expression and selected clinical parameters.

Clinical Parameter	Category	*n* (%)	Median PD-L1 Expression	*p* Value
Age	<60 years	41 (78%)	35%	0.16
	≥60 years	12 (22%)	10%	
Tumor size	<10 cm	34 (70%)	30%	0.36
	≥10 cm	14 (30%)	40%	
Serum LDH	<280 U/L	29 (67%)	40%	0.50
	≥280 U/L	14 (33%)	30%	
Serum beta-2 microglobulin	<2200 ng/mL	11 (39%)	55%	0.98
	≥2200 ng/mL	17 (61%)	30%	
Ann Arbor stage	1	4 (8%)	85%	0.55
	2	30 (60%)	30%	
	3	12 (24%)	30%	
	4	4 (8%)	50%	

Counts are based on cases with available data for each variable; percentages are calculated from non-missing cases. PD-L1, programmed death-ligand 1; LDH, lactate dehydrogenase.

## Data Availability

The data underlying this study are available from the corresponding author upon reasonable request. The data are not publicly available because they contain potentially identifiable clinical information and are subject to institutional privacy restrictions.

## Supplementary Materials

The following supporting information can be downloaded at https://www.mdpi.com/article/10.3390/diagnostics16203349/s1, Table S1: Case-level distribution of PD-L1 staining intensity in the nine mediastinal gray zone lymphoma cases.
