# Supplementary material for: PD-L1 Expression and Clinicopathologic Associations in Mediastinal Aggressive Large B-Cell Lymphomas: A Retrospective Single-Center Study
Source: Diagnostics (Basel). 2026 Oct 14;16(20):3349. doi: 10.3390/diagnostics16203349 (PMC13703436; doi:10.3390/diagnostics16203349)
Supplement: Supplementary file 1 [file diagnostics-16-03349-s001.zip › diagnostics-4541320-supplementary.pdf]

Supplementary Table S1

PD-L1 Expression and Clinicopathologic Associations in Mediastinal Aggressive Large B-Cell Lymphomas: A Retrospective Single-Center Study

Table S1. Case-level distribution of PD-L1 staining intensity in the nine mediastinal gray zone lymphoma cases.

MGZL case	0 (%)	1+ (%)	2+ (%)	3+ (%)	Overall PD-L1-positive cells (%)	3+ staining in ≥50% of evaluated cells
MGZL-01	95	5	0	0	5	No
MGZL-02	30	0	20	50	70	Yes
MGZL-03	0	10	20	70	100	Yes
MGZL-04	100	0	0	0	0	No
MGZL-05	0	0	10	90	100	Yes
MGZL-06	20	0	10	70	80	Yes
MGZL-07	100	0	0	0	0	No
MGZL-08	60	35	5	0	40	No
MGZL-09	100	0	0	0	0	No
**Group summary**	**56.11**	**5.56**	**7.22**	**31.11**	**43.89**	**4/9 (44.4%)**

• Values are percentages of all evaluated tumor cells and intratumoral inflammatory cells within the selected tumor area.
• Overall PD-L1-positive cells are the sum of the 1+, 2+, and 3+ categories.
• The strong-staining endpoint was defined a priori as 3+ staining in at least 50% of evaluated cells.
• Cases were anonymized as MGZL-01 to MGZL-09 and are presented independently of clinical outcome.

Abbreviations: MGZL, mediastinal gray zone lymphoma; PD-L1, programmed death-ligand 1.
